# Evaluation of occlusal function during orthognathic therapy: A prospective clinical trial using a digital registration method

**DOI:** 10.1007/s00056-022-00382-x

**Published:** 2022-02-28

**Authors:** Bernhard Wiechens, Anja Quast, Daniela Klenke, Phillipp Brockmeyer, Henning Schliephake, Philipp Meyer-Marcotty

**Affiliations:** 1 Department of Orthodontics, University Medical Center Goettingen, Robert-Koch-Str. 40, 37075 Goettingen, Germany; 2 Department of Oral and Maxillofacial Surgery, University Medical Center Goettingen, Robert-Koch-Str. 40, 37075 Goettingen, Germany

**Keywords:** Orthognathic surgery, Digital occlusal analysis, Occlusal efficiency, Malocclusion, Bite force, Orthognathe Chirurgie, Digitale Okklusionsanalyse, Okklusale Effizienz, Malokklusion, Bisskraft

## Abstract

**Objectives:**

Reduced occlusal function is a main characteristic of orthognathic patients. The present study aimed to investigate the extent of therapy-induced functional improvements in occlusal function using a digital diagnostic method.

**Methods:**

This prospective clinical study included 41 orthognathic patients (24 women and 17 men, median age 27.26 ± 8.2 years) and 10 control patients (5 women and 5 men, median age 29.8 ± 13.5 years) with neutral skeletal and dental configurations. The patients were divided into classes I, II and III based on their cephalometry. Digital occlusal registrations in habitual occlusion in an upright sitting position were taken before (T1) and after (T2) therapy using the T‑Scan Novus (Tekscan, South Boston, MA, USA) application.

**Results:**

Class II and III patients showed a significantly less efficient occlusal pattern than the untreated controls regarding total antagonism (*p* < 0.001), time of occlusion (*p* = 0.004), occlusal asymmetry (*p* = 0.001), anterior antagonism (*p* < 0.001) and posterior antagonism (*p* < 0.001). After therapy, the occlusal pattern increased in both therapy groups, where class III patients became indistinguishable from the controls, and class II patients differed only in posterior antagonism (*p* = 0.035).

**Conclusions:**

The digital occlusal registration method proved to be a useful diagnostic tool and provided new insights into therapeutic effects in orthognathic patients. By precisely adjusting the occlusal function, masticatory performance improved significantly.

**Clinical relevance:**

Severe malocclusion leads to a significantly lower masticatory performance for patients, which can be improved by orthognathic therapy and captured by digital occlusal registration.

## Abstract (de)

**Zielsetzung:**

Die eingeschränkte okklusale Funktion ist ein Hauptmerkmal von Dysgnathiepatient/-innen. Ziel der vorliegenden Studie war es, das Ausmaß von therapiebedingten funktionellen Verbesserungen der okklusalen Funktion mithilfe eines digitalen Registrierverfahrens zu untersuchen.

**Methoden:**

An dieser prospektiven klinischen Studie nahmen 41 Dysgnathiepatient/-innen (24 Frauen, 17 Männer, medianes Alter 27,26 ± 8,2 Jahre) und 10 Kontrollpatient/-innen (5 Frauen, 5 Männer, 29,8 ± 13,5 Jahre) mit skelettal und dental neutraler Konfiguration teil. Die Patienten/-innen wurden anhand ihrer Kephalometrie in die Angle-Klassen I, II und III eingeteilt. Digitale okklusale Registrierungen in habitueller Okklusion und aufrechter Sitzposition wurden vor (T1) und nach (T2) der Therapie unter Anwendung des T‑Scan Novus (Tekscan, South Boston, MA, USA) durchgeführt.

**Ergebnisse:**

Patienten/-innen der Angle-Klassen II und III zeigten ein signifikant weniger effizientes okklusales Muster als die unbehandelten Kontrollen in Bezug auf den Gesamtantagonismus (*p* < 0,001), die Dauer der Okklusion (*p* = 0,004), die okklusale Asymmetrie (*p* = 0,001) sowie den anterioren (*p* < 0,001) und den posterioren Antagonismus (*p* < 0,001). Nach der Therapie nahm das okklusale Muster in beiden Therapiegruppen zu, wobei sich die Patient/-innen der Klasse III nicht mehr von den Kontrollen unterschieden, während die Patient/-innen der Klasse II sich nur im posterioren Antagonismus unterschieden (*p* = 0,035).

**Schlussfolgerungen:**

Die digitale Okklusionsregistrierung erwies sich als nützliches Diagnoseinstrument und lieferte neue Erkenntnisse über die therapeutischen Auswirkungen bei Dysgnathiepatient/-innen. Durch die präzise Einstellung der okklusalen Funktion verbesserte sich die Kaufunktion signifikant.

**Klinische Relevanz:**

Schwere Zahnfehlstellungen führen bei Patient/-innen zu einer deutlich geringeren Kauleistung, die durch eine kombiniert kieferorthopädisch-kieferchirurgische Therapie verbessert und durch eine digitale Bissregistrierung erfasst werden kann.

## Introduction

The correction of malocclusion in orthognathic patients plays a key role in the rehabilitation of masticatory function [1, 2]. With an interdisciplinary team approach, orthognathic treatment leads to a recovery of occlusal function, paying particular attention to a joint-protective intervention [3, 4]. Therefore, the therapeutic objective must primarily be the rehabilitation of occlusal function in the sense of an increase in masticatory performance through a precisely adjusted dental occlusion and individually aligned jaws in proper relationship to each other [5]. Several occlusal assessment methods, such as colour-changing chewing gums, sieving of masticated food particles, scanning of masticated food particles or measuring the release of dye out of chewed boluses, have been developed to evaluate masticatory performance [6, 7]. Conversely, this means that the basis of masticatory performance can be evaluated in physiological occlusion. Currently, for the diagnosis of physiological occlusion, digital instruments can provide information about masticatory performance by utilizing quantifiable measurement parameters [8, 9]. For this purpose, digital occlusal registration allows for a quantifiable occlusal analysis and provides more information than conventional, qualitative recording methods. Thus, in addition to the number of antagonistic tooth contacts, additional parameters to describe occlusal performance have been recorded with a digital instrument [9]. These are (1) occlusal time, defined as the interval required from the initial to the final tooth contact in maximum intercuspation, (2) occlusal asymmetry, and (3) the difference in force-loaded quadrants.

Reduced tooth antagonism and/or a prolonged occlusion time resulting in reduced masticatory performance has been reported in different clinical trials [2, 10]. To the best of our knowledge, only one study exists about the assessment of occlusion using digital registration in a group of orthognathic patients [5]. To date, a detailed analysis regarding skeletal class and the extent of malocclusion has been missing in the literature. Consequently, the longitudinal impact of orthognathic therapy on the digital analysis of occlusion in different groups of orthognathic patients should be investigated.

Therefore, this study aimed

- to implement a digital registration technique in a clinical setting of interdisciplinary orthognathic treatment,
- to examine orthognathic patients differentiated by skeletal class and the extent of malocclusion longitudinally before and after treatment, and
- to quantify the therapeutically induced effects on occlusal function.

## Patients and methods

This observational cohort study with a prospective approach was approved by the local Institutional Ethics Committee of the University Medical Center Goettingen (ethics number 7/1/16). The study was carried out according to the principles of the Declaration of Helsinki and is listed in the German Clinical Trials Register. All patients participated in the trial on a voluntary basis after receiving comprehensive information about the aim and design of the study and signing an informed consent form. This report complies with the Strengthening the Reporting of Observational Studies in Epidemiology (STROBE) guidelines for observational studies [11]. A sample size of 14 patients per group was determined by a G*Power statistical analysis (v. 3.1.9.2, University of Duesseldorf) assuming a Wilcoxon signed-rank test for paired samples, with a significance level of 0.05, a power of 0.8, and a drop-out rate of 10%. The effect size was estimated according to previously reported pre- and postsurgical occlusion differences (difference: mean [M] = 3.3 teeth; standard deviation [SD] = 3.5 teeth) by Agbaje et al. [5].

### Patients

Forty-six orthognathic patients (27 women, 19 men, median age 27.4 ± 7.9 years) who received interdisciplinary treatment (orthodontics and maxillofacial surgery) participated in the trial. The patients were recruited from the Department of Orthodontics and underwent operation at the Department of Oral and Maxillofacial Surgery at the University Medical Center Göttingen. All data were collected from March 2019 to February 2021 and were analysed by a single investigator (BW). Of the 46 patients initially screened, 41 patients (24 women and 17 men, median age 27.26 ± 8.2 years) were finally included in the study (drop-out rate *n* = 5; Table 1). The drop-outs were due to lack of interest in participating in the study, change of residence, or no longitudinal follow-up for occlusal registration, which occurred when the patients were still wearing a multibracket appliance at time T2. The inclusion criteria were a severe skeletal anomaly (prognathic/retrognathic maxilla and/or mandible) with characteristic dental manifestations of an increased or inverse overjet, open bite, or deep bite. Exclusion criteria were an underlying congenital syndrome, history of cleft lip palate, history of trauma or patients who had previously been treated with an osteotomy of the maxilla/mandible.

**Table 1:** Descriptive statistics of the study patients Deskriptive Statistik der Studienpatienten/-innen

Characteristics of the study patients		Controls I (*n* = 10)		Class II (*n* = 21)		Class III (*n* = 20)	
	Unit	Median	IQR	Median	IQR	Median	IQR
Age	y.m	29.8	13.5	26.8	15.7	23.95	5.6
ANB	°	2.75	2.3	7.6	2.7	−1.3	5.3
Wits	mm	−0.6	1.3	5.6	4.3	−7.3	4.9
ML-NL	°	22.75	5.1	30.5	15.6	27.75	12.2
Overjet	mm	2.05	1.1	8.1	2.3	−1.35	3.4
Overbite	mm	1.75	1.7	1.5	2.9	0	2.3
–	–	Female	Male	Female	Male	Female	Male
Sex	*n*	5	5	15	6	9	11
First premolar agenesis	*n*	1	0	2	2	2	5
Skeletal asymmetry	*n*	0	0	5	5	8	9
Type of intervention	–	–	–	–	–	–	–
Le Fort I (solely)	*n*	0	0	0	0	0	1
BSSO (solely)	*n*	0	0	6	1	0	0
Bimaxillary	*n*	0	0	9	5	9	10

*y.m* years.months, *IQR* interquartile range; *BSSO* bilateral sagittal split osteotomy, *ANB* NA/NB-Plane angle, *ML-NL* mandibular plane and nasal plane angle

The patients were classified into two groups according to their skeletal deformity, and we included a control group with a neutral skeletal morphology.

- Class II: 21 subjects (15 females, 6 males, median age 26.8 ± 15.7 years) with an angle class II occlusion; overjet median 8.1 ± 2.3 mm and overbite median 1.5 ± 2.9 mm; Wits appraisal median 5.6 ± 4.3 mm and ML-NL (mandibular plane and nasal plane angle) median 30.5 ± 15.6°.
- Class III: 20 subjects (9 females, 11 males, median age 23.95 ± 5.6 years) with an angle class III occlusion; overjet median −1.35 ± 3.4 mm and overbite median 0 ± 2.3 mm; Wits appraisal median −7.3 ± 4.9 mm and ML-NL median 27.75 ± 12.2°.

Control group: 10 subjects (5 females, 5 males, median age 29.8 ± 13.5 years) with an angle class I occlusion; overjet median 2.05 ± 1.1 mm and overbite median 1.75 ± 1.7 mm; Wits appraisal median −0.6 ± 1.3 mm and ML-NL median 22.75 ± 5.1°. All controls had a complete dentition of natural teeth (no implants or prosthetic restorations—premolar agenesis was neither an exclusion nor an inclusion criterion). The exclusion criteria for the controls were the presence of temporomandibular disorders (TMDs), open bite, traumatic deep bite, crossbites, nonocclusions, and asymmetries.

### Methods

**Fig. 1:**
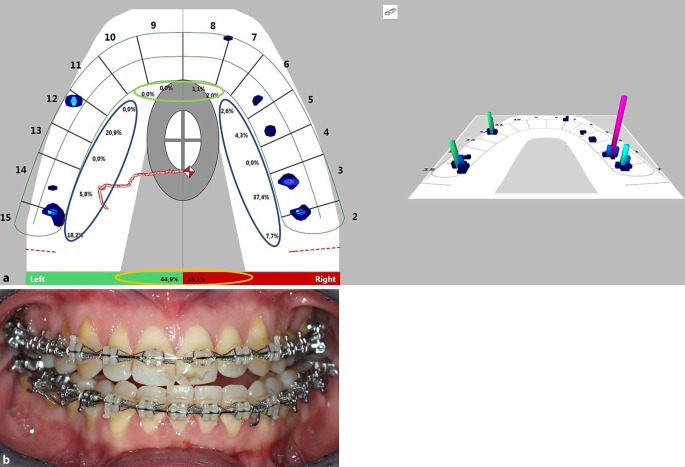
Analysis interface of the T‑Scan 9.1 software (Tekscan Inc., South Boston, MA, USA) before surgery (T1). Occlusal analysis of a class III patient (**a**) and corresponding clinical situation (**b**) before surgery (T1). Regarding the study-specific parameter, a total antagonism of 64.28% can be observed in this example. The anterior and posterior antagonism amounts to 50 and 70%, respectively, with an occlusal asymmetry of ∆ 10.2%. The quantifiably recorded occlusal findings thus illustrate the clinically imposing malocclusion (**b**) Analyseoberfläche der Software T‑Scan 9.1 (Tekscan Inc., South Boston, MA, USA) vor der Operation (T1). Okklusionsanalyse eines Patienten der Angle-Klasse III (**a**) und die entsprechende klinische Situation (**b**) vor der Operation (T1). Was die studienspezifischen Parameter betrifft, so ist in diesem Beispiel ein Gesamtantagonismus von 64,28% zu beobachten. Der anteriore und posteriore Antagonismus beläuft sich auf 50 bzw. 70%, bei einer okklusalen Asymmetrie von ∆ 10,2%. Die quantitativ erfassten okklusalen Befunde verdeutlichen somit die klinisch augenfällige Malokklusion (**b**)

**Fig. 2:**
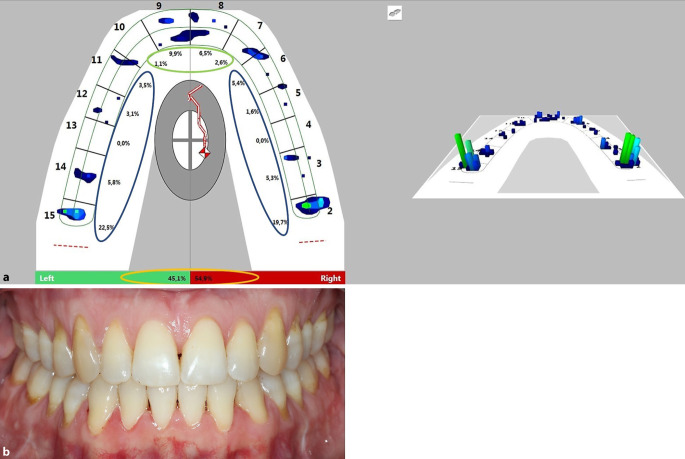
Follow-up with analysis interface of the T‑Scan 9.1 software (Tekscan Inc., South Boston, MA, USA) after therapy (T2). Occlusal analysis of the patient (**a**) and corresponding clinical situation (**b**) after therapy (T2). Regarding the study-specific parameter, a total antagonism of 85.71% was observed in the follow-up. The anterior and posterior antagonism amounts to 100 and 80%, respectively, with an occlusal asymmetry of Δ 9.8%. The quantifiably recorded occlusal findings underline the clinically apparent effect of therapeutically reintegrated occlusal support in neutral occlusion (**b**) Nachuntersuchung mit der Analyseoberfläche der T‑Scan 9.1 Software (Tekscan Inc., South Boston, MA, USA) nach der Therapie (T2). Okklusale Analyse des gleichen Patienten (**a**) und entsprechende klinische Situation (**b**) nach der Therapie (T2). Was die studienspezifischen Parameter betrifft, so wurde bei der Nachuntersuchung ein Gesamtantagonismus von 85,71% festgestellt. Der anteriore und posteriore Antagonismus beträgt 100 bzw. 80% bei einer okklusalen Asymmetrie von ∆ 9,8%. Die quantitativ erfassten okklusalen Befunde unterstreichen den klinisch sichtbaren Effekt der therapeutisch reintegrierten okklusalen Abstützung in neutraler Okklusion (**b**)

In each group, digital occlusal registration was performed using T‑Scan Novus and the associated software T‑Scan 9.1 (Tekscan Inc., South Boston, MA, USA). The system allows for occlusal registration with an electronic occlusion foil of 100 µm thickness (compressible to 64 µm). The software enables the analysis and archiving of each patient’s registration by creating an individual patient-specific virtual dental arch, as seen in Fig. 1. Therefore, the mesiodistal tooth widths of all maxillary teeth were measured for each patient separately on a model cast. For data recording, the subjects were registered in an upright sitting position in habitual occlusion. Initially, a calibration measurement with maximum bite force was registered to determine the maximum individual force. After initial bite calibration, the subjects were asked to perform one bite to the maximum possible intercuspation on command and to open their mouths again after reaching this position to complete the measurement. The result for each parameter was used for statistical analysis. The patient’s data were recorded at T1 (immediately before surgery) and at T2 after bracket removal (9 months after surgical intervention). To verify the measurement accuracy and reliability of the method, the control group was recorded twice with a minimum 1‑week interval according to the same registration procedure. The collected parameters for occlusal function were (1) total antagonism (Fig. 1 green & blue; Table 2), (2) time of occlusion (Table 2), (3) occlusal asymmetry (Fig. 1 orange ellipse; Table 2), (3) anterior antagonism, and (4) posterior antagonism (Fig. 1 green and blue, respectively; Table 2). The exact definitions of the parameters are given in Table 2. Data acquisition for occlusal registration was performed at maximum intercuspation for each registration. At this defined measurement time, all aligned teeth of the maxillary dental arch were recorded. With reference to Figs. 1 and 2, antagonism was seen whenever a percentage deflection was registered in the area of the blue and green ellipse, which could additionally be assigned a coloured bar on the dental arch graph. The height and colour of the bar visualized the percentage of the relative total force application of the respective contact point area. Occluding teeth were recorded first in total and then further in separate tooth groups: posterior (distal of the lateral incisors to distal of the second molar) and anterior (mesial to the canines; Fig. 1 blue and green, respectively; Table 2).

**Table 2:** Definitions and interpretations of the parameters Definitionen und Interpretationen der Parameter

Occlusal parameters	Unit	Definition	Interpretation
Total antagonism	%	Ratio between all teeth vs all teeth in contact × 100 Antagonistically aligned teeth of maxillary dental arch at individual maximum intercuspation (teeth in occlusion)	Antagonism was seen whenever a percentage involvement in the total force was indicated in the area of the mesiodistal boundary of the virtual tooth
Time of occlusion	s	Time from initial tooth contact to maximum intercuspation	Shortened time was seen as efficient, lengthened time as inefficient
Occlusal asymmetry ∆	%	Δ between higher and lower force-loaded quadrants	Lower values mean an increase of quadrant symmetry force-deltas (Δ) between the quadrants indicates an increase of quadrant symmetry
Anterior antagonism	%	Percentage of antagonistic aligned teeth in the frontal or posterior segment, defined from lateral to lateral incisor or as area distal of the lateral incisors	Lower percentages mean less-functional anterior antagonism
Posterior antagonism	%		Lower percentages mean less-functional posterior antagonism

### Statistics

Statistical analyses were performed using SPSS Statistics version 26 (IBM Corp., Armonk, NY, USA). The Shapiro–Wilk test was used to test the variables for normal distribution. Accordingly, none of the measurement parameters, except for the parameter “time of occlusion”, were normally distributed. For this reason, the data were subsequently analysed using the Kruskal–Wallis test for independent samples and adjusted for multiple testing using Bonferroni correction. Post hoc tests were then performed for the analysis of dependent variables between groups. Analysis of related samples was performed using Wilcoxon tests.

The significance level was set at *p* < 0.05. Study-specific measurement parameters were examined using descriptive statistics and reported as medians and interquartile ranges. To account for individual configurations, such as premolar agenesis or therapeutic premolar extractions, and to compare them with the fully edentulous study patients, a recording of antagonism in percent was chosen.

The parameters total antagonism, occlusal asymmetry, and anterior and posterior antagonism were thus output as percentages, and the associated medians and interquartile ranges were determined. In addition, the time of occlusion (analysis of occlusal velocity) was recorded, rounded to the nearest hundredth of a second, and the associated medians and interquartile ranges were also determined for each associated measurement period (Tables 3 and 4).

**Table 3:** Descriptive statistics of the occlusal parameters preoperatively (T1) according to the respective group Deskriptive Statistik der okklusalen Parameter präoperativ (T1) nach Gruppen

Occlusal parameters T1		Controls I (*n* = 10)		Class II (*n* = 21)		Class III (*n* = 20)		Overall group comparison	Intergroup comparison		
								(I vs. II vs. III)	Group I vs. II	Group I vs. III	Group II vs. III
	Unit	Median	IQR	Median	IQR	Median	IQR	*p*	Corr.*p*	Corr.*p*	Corr.*p*
Total antagonism	%	100	0	50	21.43	50	35.02	< 0.001***	< 0.001***	< 0.001***	n. s.
Time of occlusion	s	0.22	0.14	0.4	0.41	0.48	0.53	0.004**	0.017*	0.004**	n. s.
Occlusal asymmetry ∆	%	5.3	4.95	24.20	32.50	28.00	32.10	0.001**	0.006**	< 0.001***	n. s.
Anterior antagonism	%	100	37.50	0	0	37.50	50	< 0.001***	< 0.001*	0.020*	n. s.
Posterior antagonism	%	100	2.5	60	36.25	55	33.12	< 0.001***	0.001**	< 0.001***	n. s.

Controls (skeletal class I with neutral occlusion, class II: skeletal class II and angle class II occlusion, class III: skeletal class III and angle class III occlusion) and corresponding results of the Kruskal–Wallis one-way analysis of variance for the overall comparison between all groups followed by post hoc tests performed for the analysis of dependent variables between groups. The median and the interquartile range (IQR) are reported; the significance level was set at *p* < 0.05, adjusted by Bonferroni correction (corr. *p*). Kruskal–Wallis and post hoc test: *n.* *s.* not significant

* *p* < 0.05, ** *p* < 0.01, *** *p* < 0.001

**Table 4:** Descriptive statistics of the occlusal parameters after treatment (T2) according to the respective group Deskriptive Statistik der okklusalen Parameter nach der Behandlung (T2) entsprechend der jeweiligen Gruppe

Occlusal parameters T2		Controls I (*n* = 10)		Class II (*n* = 21)		Class III (*n* = 20)		Overall group comparison	Intergroup comparison		
								(I vs. II vs. III)	Group I vs. II	Group I vs. III	Group II vs. III
	Unit	Median	IQR	Median	IQR	Median	IQR	*p*	Corr.*p*	Corr.*p*	Corr.*p*
Total antagonism	%	100	0	85.71	20.84	84.52	26.19	0.014*	0.013*	n. s.	n. s.
Time of occlusion	s	0.22	0.14	0.28	0.20	0.19	0.19	n.s.	n.s.	n.s.	n.s.
Occlusal asymmetry ∆	%	5.3	4.95	17.20	32.80	16.80	20.25	n.s.	n.s.	n.s.	n.s.
Anterior antagonism	%	100	37.50	75	62.50	87.50	50	n.s.	n.s.	n.s.	n.s.
Posterior antagonism	%	100	2.5	90	25	88.75	23.75	0.034*	0.035*	n. s.	n. s.

Controls (skeletal class I with neutral occlusion; class II: skeletal class II and angle class II occlusion; class III: skeletal class III and angle class III occlusion) and corresponding results of the Kruskal–Wallis one-way analysis of variance for the overall comparison between all groups followed by post hoc tests performed for the analysis of dependent variables between groups. The median and the interquartile range (IQR) are reported; the significance level was set at *p* < 0.05, adjusted by Bonferroni correction (corr. *p*). Kruskal-Wallis and post hoc test: *n.* *s.* not significant

* *p* < 0.05

To determine the error of the occlusal registration method, the controls were recorded again, the occlusal parameters were remeasured by the same examiner after a minimum one-week interval, and the Dahlberg method [12] was applied. The determination of the method error showed that all parameters were below the reference value of 1 (range 0.007 to 0.495), which proved the validity of the method.

## Results

The demographic data for all patients, detailed clinical characteristics of the patients and controls and the type of intervention are shown in Table 1. There was no statistically significant difference between the three groups according to age (*p* = 0.071). The sex distribution in all three groups was well balanced. The results of the descriptive analysis of the occlusal parameters for all patients and controls at T1 and T2 are listed in Tables 3 and 4. The results of the Kruskal–Wallis test and the post hoc analyses are provided for the preoperative study period (T1) in Table 3 and for the postoperative period (T2) in Table 4.

At timepoint T1, the independent sample analysis for the variables revealed strong significant differences over all study groups for all analysed parameters. The intergroup analysis performed by post hoc tests also revealed significant differences in almost all parameters between both therapy groups compared to the controls.

Overall, the parameter values reflected noticeably reduced occlusal function in skeletal class II and class III patients. It was evident that both therapy groups had occlusion on only half of their teeth, which was significantly different than in the controls (total antagonism: class II/class III *p* < 0.001). In addition, the parameter “time of occlusion” was twice as long than that of the controls (class II/class III *p* < 0.001), and occlusal asymmetry was more than four times higher in class II patients (*p* = 0.006) and more than five times higher in class III patients (*p* < 0.001). With respect to anterior antagonism, the patients with skeletal class II had no contact between antagonistic teeth, resulting in insufficient anterior antagonism (*p* < 0.001). Class III patients had approximately one-third of the possible occlusion contacts (*p* = 0.020). With respect to posterior antagonism, both groups showed less than two-thirds of the possible occlusion contacts (class II/class III *p* < 0.001). In comparing the therapy groups, no significant differences could be detected for any parameter.

At timepoint T2 (9 months after surgery), all variables of the patients with skeletal class II and skeletal class III showed a clear convergence with the level of the controls. The independent samples analysis for the variables revealed significant differences concerning total and posterior antagonism. All other variables showed no significant differences when comparing the therapy groups to the controls. Post hoc analysis clarified that skeletal class III patients were indistinguishable from the control sample, whereas patients with skeletal class II still showed slightly significant differences concerning total antagonism (*p* = 0.013) and posterior antagonism (*p* = 0.035). When comparing the therapy groups, no significant differences could be seen for any of the parameters.

The Wilcoxon tests of the linked samples revealed strong significant differences for almost all parameters in both orthognathic therapy groups during the observational period of 9 months (Table 5). Only the parameter occlusal asymmetry in class II patients showed an increase in symmetry, but without statistical significance.

**Table 5:** Longitudinal overview of variables from T1-T2 concerning the combined samples. Analysis of the connected samples was performed using Wilcoxon tests. The significance level was set at *p* < 0.05 Longitudinale Übersicht der Variablen von T1-T2 in Bezug auf die kombinierten Stichproben. Die Analyse der verbundenen Stichproben wurde mittels Wilcoxon-Tests durchgeführt. Das Signifikanzniveau wurde auf *p* < 0,05 festgelegt

Occlusal parameters	Class II	Class III
	T1-T2	T1-T2
	*p*-value	
Total antagonism	< 0.001***	< 0.001***
Time of occlusion	0.010*	< 0.001***
Force symmetry ∆	n. s.	0.002**
Anterior antagonism	< 0.001***	0.001**
Posterior antagonism	< 0.001***	< 0.001***

Wilcoxon test: *n.* *s.* not significant

* *p* < 0.05, ** *p* < 0.01, *** *p* < 0.001

## Discussion

Many aspects of the benefits of orthognathic treatment for patients with severe malocclusions have been described in the literature, but only a few studies have considered longitudinal occlusal benefits. Moreover, no data about occlusal changes in a distinct patient cohort with a reliable digital occlusal registration procedure could be found. The present investigation showed a significantly less efficient occlusal function pattern in patients with severe skeletal class III malocclusion and skeletal class II malocclusion than in untreated controls at the preoperative timepoint. In contrast, after surgical/orthodontic intervention, both groups, skeletal class III and class II patients, showed changes that led to occlusal function that was very similar to that of the control group. This change in pattern analysed by a digital occlusal registration method could be seen as clear evidence for an increase in occlusal efficiency through orthognathic therapy.

The occlusal registration in this study was performed by a modern approach using a digital instrument (T-Scan Novus; T‑Scan 9.1; Tekscan Inc.). Many publications have described the clear superiority of quantitative and qualitative T‑scan procedures compared to conventional qualitative methods, primarily because they avoid the subjective interpretation of the practitioner [13]. The reliability and validity of this procedure have been the subjects of numerous studies, suggesting that it could be accepted as completely satisfactory for clinical use [14–16]. Opposing opinions about the reproducibility have emerged in the past regarding the previous generations of T‑Scans I and II, which had significantly stiffer sensor foils [17]. However, Koos et al. did not find any deficiencies in their reliability study of the T‑Scan III [15], which was also confirmed by Agbaje et al. [5]. In addition, the results of Cerna et al. [18] strengthened its reliability for recording longitudinal data, which was of particular interest for this study along with calculating antagonism in percent. Moreover, the method error in the present investigation for reliability revealed valid results for the use of the T‑Scan Novus. Therefore, it could be postulated that the applied digital data acquisition reflects a timely, reliable, and clinically easy-to-use approach for occlusal registration in orthognathic therapy patients.

The statistical analysis of the present study results indicated an inefficient masticatory function of patients with a severe skeletal malocclusion compared to controls. This was particularly evident when considering the parameters “total antagonism” and “time of occlusion”. Both treatment groups preoperatively required significantly more time from the initial to the final tooth contact in maximum intercuspation and they presented with significantly less tooth antagonism, which suggests an inefficient functional status [2, 10]. Moreover, the generally inefficient tooth contacts in the dysgnathic patients were emphasized by significantly lower values of anterior and posterior antagonism. In particular, skeletal class II patients were characterized by no contact in the anterior region from the lateral-to-lateral incisor. In contrast, class III patients showed slightly more contacts in the anterior region, which could be explained by a higher degree of dentoalveolar compensation (retrusion of the lower incisors) in the skeletal class III dysgnathic patients [19]. Although both skeletal class II and III patients are typically characterized by almost the same severe extent of skeletal malformations (seen by the Wits appraisal), in class III patients some anterior contacts are still detectable (seen by the overjet) [19].

The parameter “occlusal asymmetry” showed a significantly different bilateral occlusal pattern between controls vs. both skeletal class II and class III patients. To date, there do not exist data for orthognathic patients about possible asymmetry with a side-specific distribution of occlusal contacts. Only for adults with neutral occlusion has a side-specific asymmetry with a Δ of 5.2 in maximum static occlusion been described [20]. This is consistent with our results showing a Δ of 5.3 in the controls. In contrast, for the orthognathic patients a Δ multiplied by a factor of four (class II: ∆ 24.2) or five (class III: ∆ 28.0), which resulted in a nonhomogeneous occlusal load, was noted. In addition, the higher degree of occlusal asymmetry could be caused by skeletal and facial asymmetry in patients with severe skeletal malformations [21].

After orthognathic therapy, the results in this study showed that for almost all occlusal parameters, a clinically significant improvement could be detected for the orthognathic patients, and they showed parameter values similar to those of the control group. Patients with a skeletal class III showed consistent values for each parameter. Only patients with a skeletal class II showed a persistent significant difference for the parameter “total antagonism” at timepoint T2, which, according to the post hoc test, was primarily attributable to deviations in posterior antagonism. This could result from the fact that one-third of the class II subjects were treated with isolated mandibular advancement, whereas in the class III patients, almost all of them were treated with bimaxillary surgery.

Regarding the impact of orthognathic therapy on asymmetries, the existing literature refers primarily to skeletal or soft tissue effects [22]. Therefore, no reliable studies that dealt with the occlusal effects of orthognathic surgical rehabilitation for comparison were identified. At T2 (Table 4), no significant difference was observed for occlusal asymmetry in either class II or class III patients compared to the control group. Thus, we conclude that therapy-induced rehabilitation of asymmetry at the tooth level can be established.

In reviewing the study results and considering that the occlusal registrations were performed by only one clinically experienced examiner, the results can be accepted as valid and conclusive to describe the effects of orthognathic therapy. Above all, the changes in the parameter “occlusal time”, which had not been determined previously, highlight the diagnostic value on the one hand and the therapeutic value of the orthodontic/surgical therapy on the other. Based on the reduced occlusion time and the increase in total antagonism in both therapy groups, the enhancement in occlusal function resulting in an increase in masticatory efficiency was shown.

## Conclusion

Digital occlusal registration could be clinically implemented with an easy and fast approach in orthognathic therapy. Digital occlusal registration proved to be a useful diagnostic tool and provided new insights into therapeutic effects. Patients with severe malocclusion are characterized by inefficient occlusal function compared to controls and develop increased masticatory efficiency after orthognathic therapy.
